# The Modeling and Simulation of the Galvanic Coupling Intra-Body Communication via Handshake Channel

**DOI:** 10.3390/s17040863

**Published:** 2017-04-14

**Authors:** Maoyuan Li, Yong Song, Wansong Li, Guangfa Wang, Tianpeng Bu, Yufei Zhao, Qun Hao

**Affiliations:** School of Optoelectronics, Beijing Institute of Technology, Beijing Key Laboratory for Precision Optoelectronic Measurement Instrument and Technology, Beijing 100081, China; 2932183@bit.edu.cn (M.L.); walsonlee1991@163.com (W.L.); zxcvbmn2009@126.com (G.W.); butianpeng@outlook.com (T.B.); zhaoyufei@bit.edu.cn (Y.Z.); qhao@bit.edu.cn (Q.H.)

**Keywords:** intra-body communication, galvanic coupling, transfer function, Information interaction

## Abstract

Intra-body communication (IBC) is a technology using the conductive properties of the body to transmit signal, and information interaction by handshake is regarded as one of the important applications of IBC. In this paper, a method for modeling the galvanic coupling intra-body communication via handshake channel is proposed, while the corresponding parameters are discussed. Meanwhile, the mathematical model of this kind of IBC is developed. Finally, the validity of the developed model has been verified by measurements. Moreover, its characteristics are discussed and compared with that of the IBC via single body channel. Our results indicate that the proposed method will lay a foundation for the theoretical analysis and application of the IBC via handshake channel.

## 1. Introduction

Intra-body communication (IBC) is a technology using the conductive properties of the human body to transmit signals [1], thus providing a novel communication method among wearable sensors in body sensor networks (BSN) [2,3,4]. Compared with current short-distance wireless technologies, such as Bluetooth and Zigbee, IBC technology has the advantages of high reliability, high speed, low consumption, and security [5,6,7]. As a result, real-time biomedical monitoring can be achieved by surface-mounted and implantable biomedical devices using IBC technology [7]. The other important application of IBC technology is that two individuals can exchange data by shaking hands without complicated operations. In this application, when two individuals shake hands, one individual can automatically acquire the other’s electric business cards through the handshake channel. Moreover, IBC technology also can be used for transferring multimedia data, creating human ad-hoc networks, and providing an assistance system for handicapped people [8,9].

In the literature, the scenario of IBC via handshake channel originated with Zimmerman [10]. Then, Shinagawa et al. designed a transceiver based on the electro-optic effect, and carried out an IBC experiment involving signal transmission via shaking hands [5]. Further to this, Hachisuka et al. measured signal transmission gains between two persons by shaking hands [11]. Shimamoto et al. measured the differences in received signal power attenuation between two individuals who touched, compared to two individuals who did not touch [8]. It should be noted that the above works were analyzed using the model of IBC via a single body channel, rather than the model of the IBC via handshake channel. Actually, the model of the IBC via handshake channel is very important for determining its characteristics, which will help to guarantee the safety of human bodies as well as the design of its application system [12,13,14,15]. However, it is generally not easy to develop a mathematical model galvanic coupling intra-body communication via handshake channel. Firstly, compared with IBC models via a single body channel, models of this kind of IBC need to include not only two or more human parts, but also the contacting parts between the two individuals, resulting in a comparatively complex structure. Secondly, due to the fact that more factors (such as contact parts, contact area, contact method, human body posture and contact pressures) will affect its characteristics, more parameters need to be considered in its mathematical modeling, while some of the parameters are difficult to define. Due to the reasons mentioned above, only the modeling of the IBC via a single body channel has been reported [16,17,18,19,20,21,22], few works refer to the modeling of the IBC via handshake channels, and its characteristics remain undisclosed so far.

In this paper, we investigate the modeling of galvanic coupling intra-body communication via handshake channels using the transfer function method. Firstly, we propose a circuit model of the galvanic coupling IBC via handshake channel [23]. Secondly, the modeling process of the contacting parts between two individuals is discussed in detail. Furthermore, the corresponding mathematical model of the whole IBC system is developed. Finally, the validity of the developed model is verified by measurements. The characteristics of the galvanic coupling IBC via handshake channel are compared and discussed with that of the IBC via a single body channel. Our results indicate that the proposed method will lay a foundation for the theoretical analysis and application of the IBC via handshake channel.

## 2. Circuit Model

### 2.1. The Whole Circuit Model

**Figure 1:**
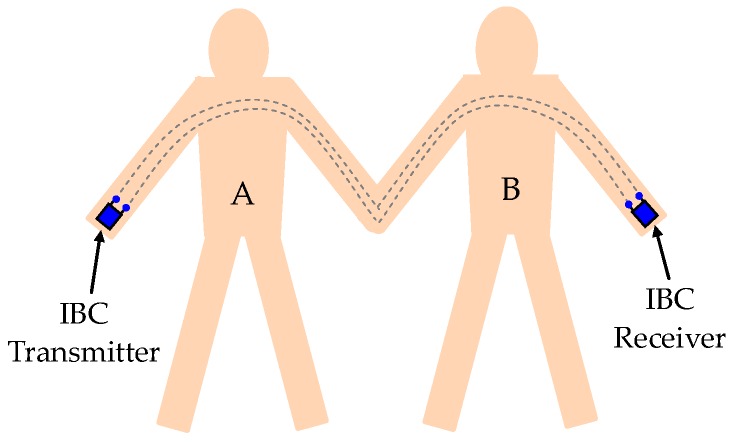
The galvanic coupling intra-body communication between subject A and B via handshake channel.

In the galvanic coupling intra-body communication via handshake channel [20], as shown in Figure 1, when two individuals (subject A and B) shake hands; the IBC transmitter attached to subject A detects a triggering signal, prompting it to output digital data through a pair of differential electrodes. The data signal transmits through subject A, the contacting part of the two hands, and subject B. Finally, it is detected by the IBC receiver attached to subject B. Therefore, the galvanic coupling intra-body communication between subject A and B can be achieved by shaking hands.

**Figure 2:**
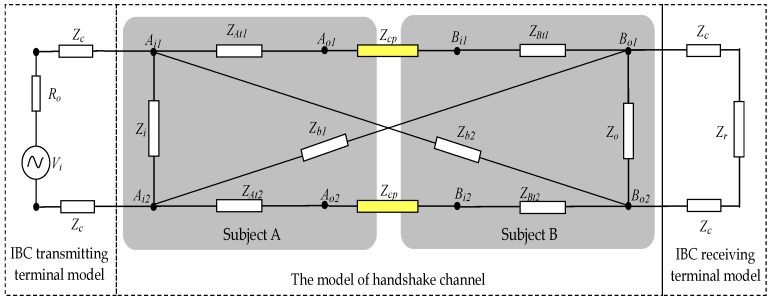
The circuit model of the galvanic coupling intra-body communication via handshake channel.

The circuit model of the galvanic coupling IBC via handshake channel is developed by a four-terminal circuit model with discrete complex impedances [10], as shown in Figure 2, which includes the IBC transmitting terminal model, the model of the handshake channel, and the IBC receiving terminal model.

In the IBC transmitting terminal model, as shown in Figure 2, *V_i_* represents a voltage source, *R_o_* is the internal resistance of the IBC transmitter, and *Z_c_* represents the coupling impedances between the transmitting electrodes and the skin of subject A.

The model of the handshake channel includes the circuit models of subject A, the contacting parts corresponding to the shaking hands, and subject B, respectively. In the circuit models of subject A and B, *Z_i_* and *Z_o_* are the input impedance and output impedance of the whole channel, respectively. *Z_b_*_1_ and *Z_b_*_2_ are the cross impedances of the whole channel. On the other hand, *Z_At_*_1_, *Z_At_*_2_ represent the transverse impedances of subject A, while the transverse impedances of subject B are represented as *Z_Bt_*_1_ and *Z_Bt_*_2_. Meanwhile, *Z_cp_* represents the contact impedances corresponding to the contacting parts caused by shaking hands, which are very important for the whole circuit model. In the model of the handshake channel, the body impedances except for contact impedances are approximately equivalent to parallel circuits of resistance and capacitance, where parameters are up to Cole-Cole equation [24].

In the IBC receiving terminal model, *Z_c_* represents the coupling impedances between the receiving electrodes and the skin of subject B, and *Z_r_* represents the input impedance of the receiver in the IBC receiving terminal model [23,25].

### 2.2. The Contacting Parts Corresponding to Shaking Hands

**Figure 3:**
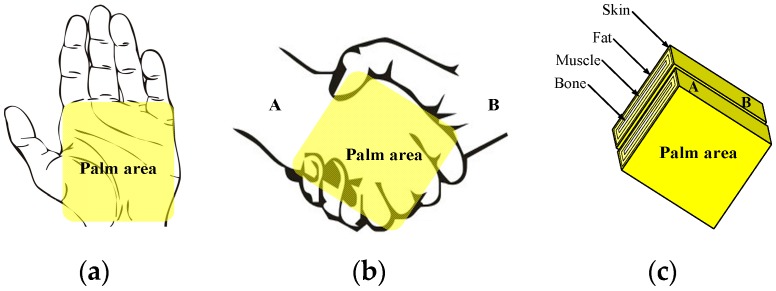
Modeling of contacting parts corresponding to shaking hands, (**a**) human hand, (**b**) handshake gesture, and (**c**) the modeled palm corresponding to handshake.

As shown in Figure 3a, the hand of the human body can be divided into palm and fingers. Generally, in the case of shaking hands, the palms of two hands are in close contact with one another, and the palm area is the main area of contact between the two hands. Even if the fingers of one hand also make contact with the back of the other hand, the contact area is comparatively small, and the contact is slight, which can be ignored. As shown in Figure 3b, the contact area has been simplified as two palms in contact with each other for the purposes of our modeling. As shown in Figure 3c, the contacting parts were abstracted as two multi-layer cuboids in contact with each other and which have approximately the same dimensions as palms A and B. Meanwhile, we assumed the cuboids consist of the layers of skin, fat, muscle, cortical bone, and bone marrow [26,27], and that their impedances could be calculated according to their conductivities and relative permittivity [24], as well as the geometric parameters of the palm model, in which the tissue of palm has the same percentage as that of arm.

**Figure 4:**
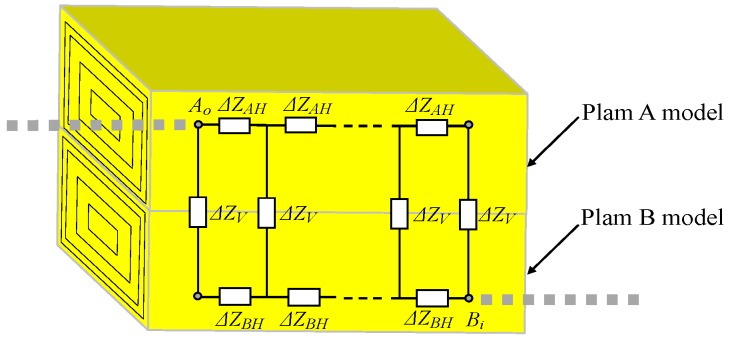
The circuit model of the contacting parts corresponding to shaking hands.

Based on the geometric modeling described above, the circuit model of *Z_cp_* can be derived. As shown in Figure 4, the circuit model of *Z_cp_* is abstracted as an impedance network, which consists of a number of unit transverse impedances (Δ*Z_H_*) and unit longitudinal impedances (Δ*Z_V_*). Of these, Δ*Z_AH_* and Δ*Z_BH_* represent the transverse impedance per unit length in the palm A model and the palm B model, respectively, while Δ*Z_V_* represents the longitudinal impedances per unit length of the whole model of palm A and palm B. Additionally, there is a containment relationship between the circuit model of Figure 2 and that of Figure 4. The nodes of *A_o_* and *B_i_* in Figure 4 correspond to *A_o_*_1_/*A_o_*_2_ and *B_o_*_1_/*B_o_*_2_ in Figure 2, respectively.

*A_o_* is the signal input/output point between the subject A model and the palm A model, while *B_i_* is the signal input/output point between the palm A model and the subject B model.

#### 2.2.1. Transverse Impedance *Z_H_*

**Figure 5:**
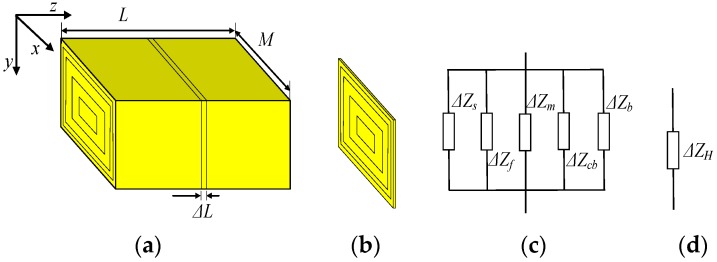
Determination of *Z_H_*, (**a**) the modeled plam, (**b**) the modeled palm with unit length, (**c**) the unit impedances corresponding to five-layer tissue, and (**d**) the unit impedance Δ*Z_H_*.

As shown in Figure 5a, the geometry of the palms is modeled by a cuboid with five layers, including skin, fat, muscle, cortical bone and bone marrow. It is assumed that *L* and *M* represent the length and width of the cuboid, respectively, while Δ*Z_H_* represents the unit transverse impedance with the unit length of Δ*L*, as shown in Figure 5b. Due to the parallel form of the five-layer tissue shown in Figure 5b, the unit transverse impedance (Δ*Z_H_*) can be expressed as the parallel connection of the impedances corresponding to the five layers, which include the impedances of the layers of the skin (Δ*Z_s_*), fat (Δ*Z_f_*), muscle (Δ*Z_m_*), cortical bone (Δ*Z_cb_*) and bone marrow (Δ*Z_b_*), respectively. Finally, the unit transverse impedance (Δ*Z_H_*) can be determined according to the following equation, which is deduced from the circuit model shown in Figure 5c.

(1) $$\Delta Z_{AH}=\frac{1}{\sum_{l=1}^{5}\frac{1}{\Delta Z_{l}}}=\frac{1}{\sum_{l=1}^{5}\left(\frac{1}{R_{l}}+j\omega C_{l}\right)}=\frac{\Delta L}{\sum_{l=1}^{5}\sigma_{lf}S_{l}+j\omega \varepsilon_{0}\sum_{l=1}^{5}\varepsilon_{rlf}S_{l}}$$

where *S_l_* is the cross-sectional area of the *l*th layer. *σ_lf_* and *ε_rlf_* are the conductivity and relative permittivity corresponding to the different layers and signal frequencies, respectively.

#### 2.2.2. Longitudinal Impedance *Z_V_*

**Figure 6:**
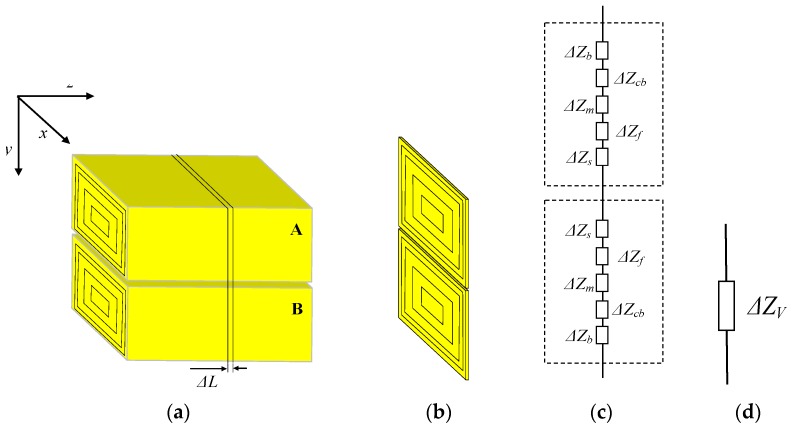
Determination of *Z_V_*; (**a**) the modeled plams corresponding to handshake, (**b**) the modeled palm with unit length, (**c**) the unit impedances corresponding to five-layer tissue, and (**d**) the unit impedance Δ*Z_V_*.

As shown in Figure 6a, the contacting parts corresponding to the handshake are modeled by two cuboids that are in contact with each other. Meanwhile, Δ*Z_V_* represents the unit longitudinal impedance, with a unit length of Δ*L*, as shown in Figure 6b. On the other hand, the models of palm A and the five-layer tissue can be regarded as a series connection of the five tissue layers along the *y-*axis. Therefore, both the unit transverse impedance of palm A and that of palm B can be expressed as a series connection of the five tissue layers. As a result, both Δ*Z_VA_* and Δ*Z_VAB_* are equal to the sum of Δ*Z_s_*, Δ*Z_f_*, Δ*Z_m_*, Δ*Z_cb_* and Δ*Z_b_*, as shown in Figure 6c. Finally, Δ*Z_VA_* and Δ*Z_VB_* can be determined according to Equation (2), and the unit longitudinal impedance (Δ*Z_V_*) of the model shown in Figure 6d is equal to the sum of Δ*Z_VA_* and Δ*Z_VB_*.

(2) $$\Delta Z_{VA}=\sum_{l=1}^{5}\frac{1}{\Delta Z_{l}}=\sum_{l=1}^{5}\left(\frac{1}{R_{l}}+j\omega C_{l}\right)=\frac{\sum_{l=1}^{5}\sigma_{lf}S_{l}+j\omega \varepsilon_{0}\sum_{l=1}^{5}\varepsilon_{rlf}S_{l}}{\Delta L}.$$

## 3. Mathematical Model

### 3.1. The Transfer Function

**Figure 7:**
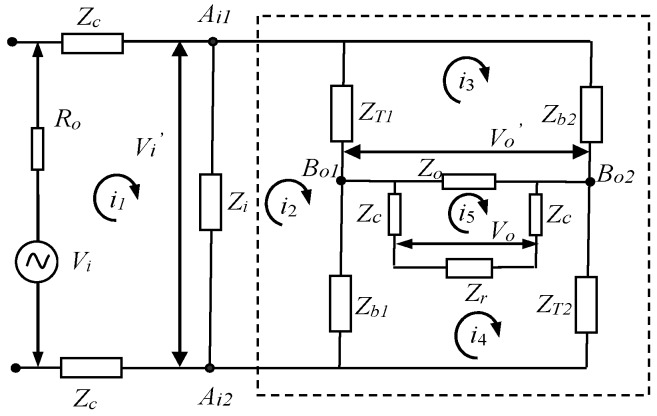
The equivalent circuit of the galvanic coupling IBC via handshake channel.

Based on the proposed circuit model, a mathematical model of a galvanic coupling IBC can be obtained. The equivalent circuit of the proposed circuit model can be developed using the method of equivalent transformation, as shown in Figure 7.

According to Figure 2, the series impedance *Z_t_*_1_, *Z_cp_*, and *Z_t_*_1’_ can be equivalent to *Z_T_*_1_ shown in Figure 7, which is the sum of *Z_t_*_1_, *Z_cp_*, and *Z_t_*_1’_; while the series impedance *Z_t_*_1_, *Z_cp_* and *Z_t_*_1’_ can be equivalent to *Z_T_*_1_ shown in Figure 7. Therefore, the following equation can be achieved.

(3) $$\left\{ \begin{array}{l} Z_{T1}=Z_{At1}+Z_{cp}+Z_{Bt1}\\ Z_{T2}=Z_{At2}+Z_{cp}+Z_{Bt2} \end{array}\right.$$

The transfer function can be expressed by five linear equations, which can be calculated by a matrix form, as shown as Equation (4).

(4) $$\left[\begin{array}{l} V_{i}\\ 0\\ 0\\ 0\\ 0 \end{array}\right]=\left[\begin{array}{ccccc} 2Z_{c}+Z_{i}+R_{0} & -Z_{i} & 0 & 0 & 0\\ -Z_{i} & Z_{i}+Z_{T1}+Z_{b1} & -Z_{T1} & -Z_{b1} & 0\\ 0 & -Z_{T1} & Z_{T1}+Z_{b2}+Z_{o} & 0 & -Z_{o}\\ 0 & -Z_{b1} & 0 & Z_{T2}+Z_{b2}+2Z_{c}+Z_{r} & -2Z_{c}-Z_{r}\\ 0 & 0 & -Z_{o} & -2Z_{c}-Z_{r} & Z_{o}+Z_{r}+2Z_{c} \end{array}\right]\left[\begin{array}{l} i_{1}\\ i_{2}\\ i_{3}\\ i_{4}\\ i_{5} \end{array}\right]$$

The matrix multiplication form corresponding to Equation (4) is

(5) V=ZI

where **V** is a column vector, in which the elements represent independent voltage source in each mesh, **Z** is a 5 × 5 impedance matrix, and **I** is a column vector holding mesh currents. Thus, the values of **I** can be obtained by multiplying the voltage vector by the inverse matrix of **Z**, and

(6) $$I=Z^{-1}V$$

Therefore, the value of output voltage *V_o_* can be calculated as

(7) $$V_{o}=(i_{4}-i_{5})Z_{r}$$

On the basis of Equation (7), the transfer function *H* can be obtained,

(8) $$H=\frac{\left|V_{o}\right|}{V_{i}}$$

Therefore, the whole transfer function can be expressed as Equation (9),

(9) $$\{\begin{array}{l} \begin{array}{l} H=\frac{H_{h}Z_{r}Z_{i}Z_{h}}{\left(Z_{r}+2Z_{c}\right)\left[\left(Z_{i}Z_{h}+\left(2Z_{c}+R_{0}\right)(Z_{i}+Z_{h})\right)\right]}\\ Z_{h}=\frac{Z_{T1}\left(Z_{T2}Z_{o}^{\prime }+Z_{b2}Z_{o}^{\prime }\right)}{\left(Z_{T1}+Z_{b2}+Z_{o}^{\prime }-kZ_{o}^{\prime }\right)\left(Z_{o}^{\prime }-Z_{T2}H_{h}\right)} \end{array}\\ H_{h}=\frac{(k-1)Z_{o}^{\prime }}{Z_{b2}+kZ_{T2}}\\ \begin{array}{l} k=\frac{Z_{b1}Z_{T1}+Z_{b1}Z_{o}^{\prime }+Z_{b1}Z_{b2}+Z_{o}^{\prime }Z_{T1}}{Z_{b1}Z_{T1}+Z_{b1}Z_{o}^{\prime }+Z_{T2}Z_{T1}+Z_{o}^{\prime }Z_{T1}}\\ Z_{T1}=Z_{At1}+Z_{Bt1}+Z_{cp}\\ Z_{T2}=Z_{At2}+Z_{Bt2}+Z_{cp}\\ Z_{o}^{\prime }=Z_{o}//\left(2Z_{c}+Z_{r}\right) \end{array} \end{array}$$

Equation (9) shows the whole transfer function of galvanic coupling intra-body communication via handshake channel, which includes the transmitter, subject A, the contacting parts corresponding to shaking hands, subject B, and the receiver, and can simulate the whole signal transmission for this kind of IBC. In Equation (9), *H* represents the whole transfer function; *Z_h_* represents the total impedance of dotted portion shown in Figure 7; *H_h_* represents the transfer function of the circuit between *V_i_’* and *V_o_’*; *k* is the ratio of *i*_4_ to *i*_3_; *Z_T_*_1_ is an equivalent series impedance formed by *Z_t_*_1_, *Z_cp_*, *Z_t_*_1_*’*; *Z_T_*_2_ is an equivalent series impedance formed by *Z_t_*_2_, *Z_cp_*, *Z_t_*_2_*’*; and *Z_o_’* is the impedance corresponding to the *V_i_’* between nodes *B_o_*_1_ and *B_o_*_2_.

Finally, the attenuation of the signal transmission in the galvanic coupling IBC via handshake channel can be determined by

(10) $$G=20\log_{10}H+K$$

where *K* is the correction factor used for correcting the inherent error between the measurements and the simulations. Generally, *K* is influenced by the modeling method, the parameter determination method and the measurement precision [23]. A detailed discussion of *K* is provided in Section 4.2.

### 3.2. Determination of Z_cp_

**Figure 8:**
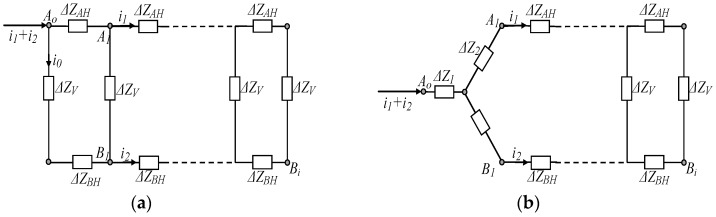
The determination of *Z_cp_*. (**a**) is the analysis of the circuit model shown in Figure 4; (**b**) is the equivalent circuit of (**a**).

To determine the value of *Z_cp_*, the circuit model shown in Figure 4 should be defined. Figure 8a shows the determination method of *Z_cp_*, and Figure 8b is the equivalent circuit of Figure 8a. As illustrated in Figure 8, if voltage is applied at the nodes of *A*_1_, *B*_1_ and *A_o_*, the corresponding currents of *i*_1_, *i*_2_, and *i*_1_+*i*_2_ can be generated. According to the circuit shown in Figure 8a, the following equation can be obtained.

(11) $$\left(\Delta Z_{V}+\Delta Z_{BH})i_{0}=\Delta Z_{AH}(i_{1}+i_{2}-i_{0})+\Delta Z_{V}(i_{2}-i_{0}\right)$$

As a result, the current of *i*_0_ can be expressed as

(12) $$i_{0}=\frac{\Delta Z_{AH}i_{1}+(\Delta Z_{AH}+\Delta Z_{V})i_{2}}{\Delta Z_{AH}+\Delta Z_{BH}+2\Delta Z_{V}}$$

On the other hand, according to the Kirchhoff’s circuit laws, the following equation can be obtained.

(13) $$\left\{ \begin{array}{l} u_{A_{o}B_{1}}=(\Delta Z_{V}+\Delta Z_{BH})i_{0}\\ u_{A_{o}A_{1}}=\Delta Z_{AH}(i_{1}+i_{2}-i_{0}) \end{array}\right.$$

Substitute *i*_0_ into Equation (13), leading to the following equation:

(14) $$\left\{ \begin{array}{l} u_{A_{o}B_{1}}=\frac{\Delta Z_{AH}(\Delta Z_{V}+\Delta Z_{BH})i_{1}+(\Delta Z_{V}+\Delta Z_{BH})(\Delta Z_{AH}+\Delta Z_{V})i_{2}}{\Delta Z_{AH}+\Delta Z_{BH}+2\Delta Z_{V}}\\ u_{A_{o}A_{1}}=\frac{\Delta Z_{AH}(\Delta Z_{BH}+2\Delta Z_{V})i_{1}+\Delta Z_{AH}(\Delta Z_{BH}+\Delta Z_{V})i_{2}}{\Delta Z_{AH}+\Delta Z_{BH}+2\Delta Z_{V}} \end{array}\right.$$

Meanwhile, according to the circuit shown in the Figure 8b, the following equations can be set up,

(15) $$\left\{ \begin{array}{l} u_{A_{o}B_{1}}=\Delta Z_{1}i_{1}+(\Delta Z_{1}+\Delta Z_{3})i_{2}\\ u_{A_{o}A_{1}}=(\Delta Z_{1}+\Delta Z_{2})i_{1}++\Delta Z_{1}i_{2} \end{array}\right.$$

According to Equations (14) and (15), the following equation can be obtained.

(16) $$\left\{ \begin{array}{l} \Delta Z_{1}=\frac{\Delta Z_{AH}(\Delta Z_{V}+\Delta Z_{BH})}{\Delta Z_{AH}+2\Delta Z_{V}+\Delta Z_{BH}}\\ \Delta Z_{2}=\frac{\Delta Z_{AH}\Delta Z_{V}}{\Delta Z_{AH}+2\Delta Z_{V}+\Delta Z_{BH}}\\ \Delta Z_{3}=\frac{\Delta Z_{V}(\Delta Z_{V}+\Delta Z_{BH})}{\Delta Z_{AH}+2\Delta Z_{V}+\Delta Z_{BH}} \end{array}\right.$$

Finally, the value of *Z_cp_*, which represents the contact impedance of the contacting parts corresponding to the shaking hands, can be determined by loop processes.

## 4. Measurements and Analysis

### 4.1. Measurement Setup

An in vivo measurement setup was built to verify the feasibility of the developed mathematical model. Meanwhile, the amplitude-frequency characteristics of the galvanic coupling IBC via handshake channel were carried out.

**Figure 9:**
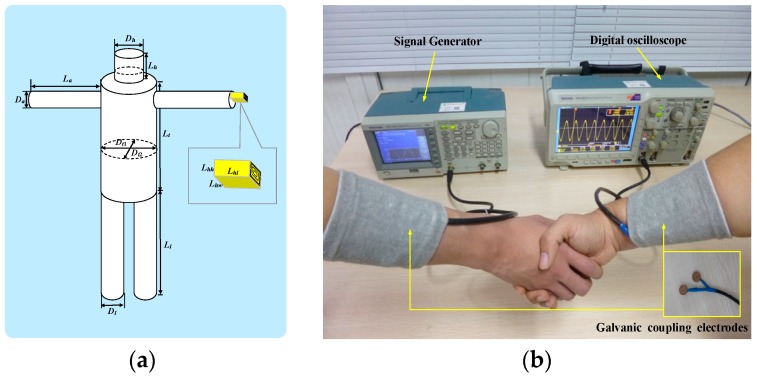
Parameter definitions and measurement setup. (**a**) Definitions of the geometry parameters; (**b**) The measurement setup.

In our experiments, two males were chosen as the subjects, whose geometry parameters are shown in Table 1, the definitions of the geometry parameters are shown in Figure 9a. The measurement setup was composed of a signal generator (Tektronix AFG3101, *R*_0_ = 50 Ω), a digital oscilloscope (Tektronix MSO3012, *Z_r_* = 1 MΩ), the galvanic coupling electrodes and two high-power lithium power batteries, as shown in Figure 9b. In our measurements, the galvanic coupling electrodes included two pairs of circular copper electrodes with the radius of 10 mm.

Considering the geometry of the arm, both the transmitter electrode and the receiver electrode were attached to the arms at a distance of 6 cm [27]. Moreover, 10 in vivo measurements were conducted within 10 days to eliminate the influence of time, with the average attenuations being considered to be the measured values in our experiment. In addition, the signal generator output sinusoidal signals had an amplitude of 5 V (peak-to-peak value), which is below the safety thresholds and avoids resulting in adverse heating effects [19]. The frequency range of 100 kHz–6 MHz was selected, for which the lower boundary is well above the spectrum of biological signals of the human body. Meanwhile, according to [18], the lumped circuit model is generally valid only when *Lc* < *λ*, where *Lc* is the circuit’s characteristic length, and *λ* is the circuit’s operating wavelength. For IBC via handshake channel, due to the fact that two subjects are involved in the signal transmission channel, the dimensions (the circuit’s characteristic length) of the IBC via handshake channel is about double that of the IBC via single body channel, which indicates that the operated frequency should be cut in half. Therefore, the upper limit frequency should be set at approximate half of the upper limit frequency (10 MHz) of the IBC via single body channel. As a result, the signal electromagnetic field transmitting within two human bodies can be regarded as a near-field region, which can be modeled using a static circuit model. Finally, the upper limit frequency was set at 6 MHz in our experiments. Additionally, the influence corresponding to the operated frequency between the measurement instruments and the cables was ignored [28,29,30].

### 4.2. Signal Transmission of the Different Distances

**Figure 10:**
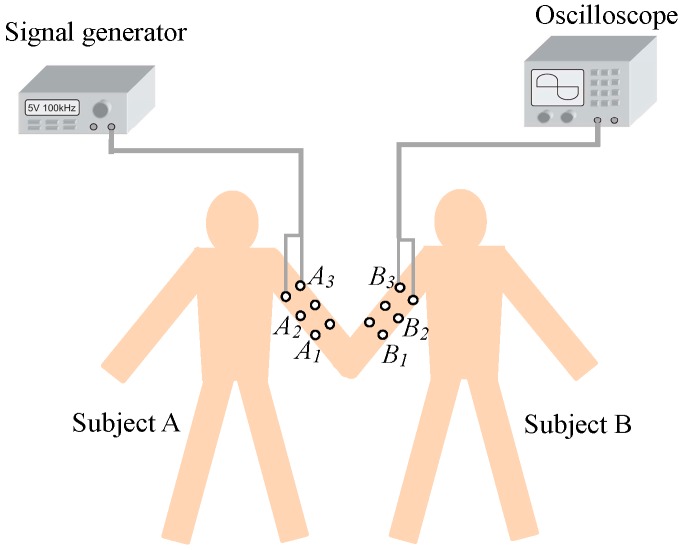
The in vivo measurements of the different signal transmission distances.

The in vivo measurements along the arms and hands of the two subjects who were shaking hands were carried out. As shown in Figure 10, the transmitting electrodes were placed on the positions of *A*_1_, *A*_2_ and *A*_3_ of subject A, while the receiving electrodes were placed on *B*_1_, *B*_2_, and *B*_3_ of subject B, resulting in signal transmission distances of 30 cm (*A*_1_–*B*_1_), 40 cm (*A*_2_–*B*_2_) and 50 cm (*A*_3_–*B*_3_), respectively. Additionally, according to our measurement experiments, the tiny gap between the two hands had a small influence on the signal attenuation of the IBC via handshake channel. In our subsequent experiments, in order to unify the experimental conditions, the two hands of the two subjects contacted tightly in the processes of shaking hands, and the influence of the tiny gap between the two hands was ignored.

Meanwhile, on the basis of the signal transmission distances, electrode size, and geometry parameters of the subjects, the corresponding simulations were carried out using the developed mathematical model. Table 2 shows some of the parameters used in the simulation, which includes *Z_c_*, *Z_At_*_1_/*Z_At_*_2_, *Z_Bt_*_1_/*Z_Bt_*_2_, *Z_i_*, *Z_o_*, *Z_cp_*, *R*_0_ and *Z_r_*, where *L* represents the distance between the electrodes of TX and RX.

**Figure 11:**
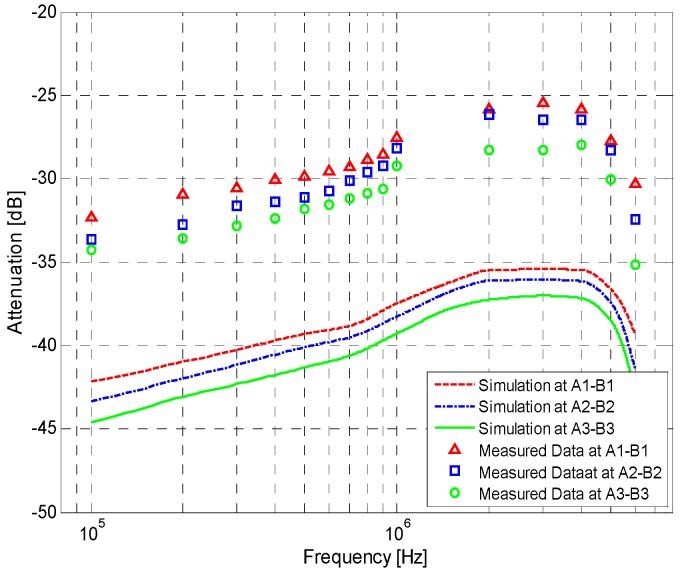
Simulation results and the measurement results of the different signal transmission distances without the correction factor.

In our investigation, due to the influences of the simulation method, the parameter determinations and the measurement setup’s precision, there is an inherent error between measurement and simulation. As shown in Figure 11, an approximate constant error with a standard deviation of 0.41 dB can be found between measurement results and the corresponding simulation results, which is similar to the case of the IBC via single body channel [23]. In the conventional simulation of the IBC via handshake channel, in order to eliminate this inherent error, a correction factor of *K* is used, which is equal to the average of the errors (9.36 dB). If higher precision is needed, the *K* corresponding to the IBC system should be calibrated. In concrete terms, the measurement should first be carried out under the condition that two individuals are shaking hands. Then, the corresponding simulation should be carried out using the proposed method. Finally, the *K* corresponding to the IBC system can be determined by calculating the differences between the measurement and simulations.

**Figure 12:**
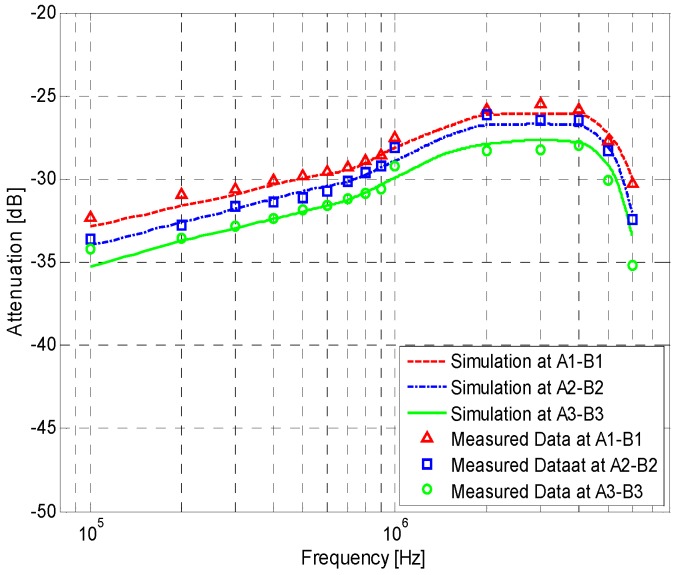
Simulation and measurement results of the signal transmission with different distances.

The signal attenuations of the measurement and simulation results are shown in Figure 12. The average and standard deviation of the signal at *A*_1_–*B*_1_ over 10 days are shown in Table 3.

According to the results shown in Figure 12, some observations can be deduced as follows: (1) The mathematical simulation results basically agree with the corresponding in vivo measurement results. For instance, with a signal frequency of 100 kHz–2 MHz, the average errors between simulations and measurements are only 0.12 dB (*A*_1_–*B*_1_), 0.26 dB (*A*_2_–*B*_2_) and 0.48 dB (*A*_3_–*B*_3_), respectively; (2) Both the simulation results and the in vivo measurement results decrease as the signal frequency increases from 100 kHz to 2 MHz. In a frequency range of 2 MHz–4 MHz, both of them reach their minimum values and remain almost unvaried. As the signal frequency increases from 4 MHz to 6 MHz, the simulation and measurement results increase rapidly; (3) As the transmission distance increases, a corresponding increase of the signal attenuations in the measurement and simulation results can also be found. Specifically, an increase of 10 cm in the signal transmission distance leads to an extra average attenuation of 1.11 dB in the simulation results, while the corresponding value in the measurement results is 1.20 dB.

### 4.3. Signal Transmission within the Different Human Parts

**Figure 13:**
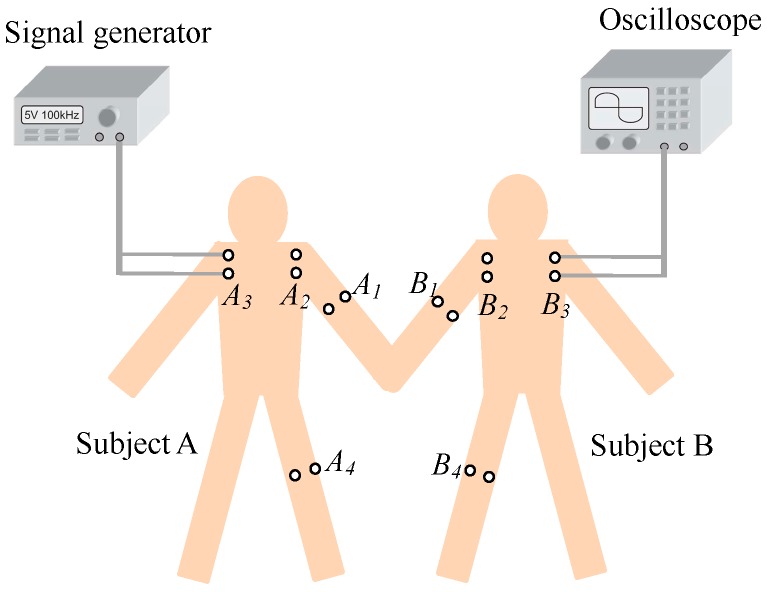
The in vivo measurements of signal transmission between different body parts of the two subjects.

In the galvanic coupling IBC via handshake channel, IBC devices may be placed on different parts of the body, such as the arm, torso and leg. As a result, signal may be transmitted along different paths within the two human bodies. As shown in Figure 13, simulation and in vivo measurements for different signal transmission paths within two subjects who were shaking hands were carried out, in which the IBC electrodes were placed on the upper arm (*A*_1_, *B*_1_), torso (*A*_2_, *A*_3_, *B*_2_, *B*_3_) and thigh (*A*_4_, *B*_4_) of the two subjects. The distances of the four paths were 60 cm (*A*_1_–*B*_1_), 120 cm (*A*_2_–*B*_2_), 180 cm (*A*_3_–*B*_3_) and 240 cm (*A*_4_–*B*_4_), respectively.

**Figure 14:**
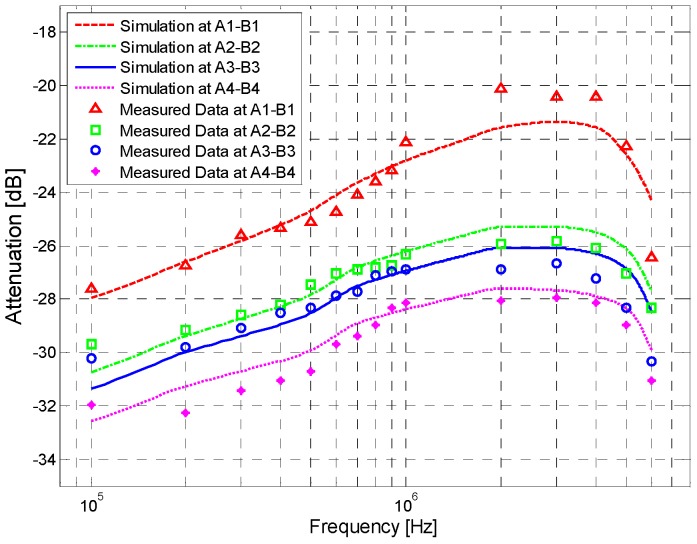
Simulation and measurement results of signal transmission within different body parts.

Figure 14 shows the simulation and measurement results of the signal transmission between the different body parts. According to Figure 14, some observations can be deduced as follows: (1) the mathematical simulation results coincide with the measurement results; (2) all of the signal attenuation curves corresponding to the different body parts have similar outlines; (3) both the measurement and that of the simulation increase with the increment of the transmission distance.

In addition, according to Figure 14, signal transmission paths of the different human parts influence the signal attenuations to some extent. For instance, the extra average attenuation of the *A*_2_–*B*_2_ path (120 cm) increased by 3.48 dB (measurement) and 3.62 dB (simulation) compared with that of the *A*_1_–*B*_1_ (60 cm) path. Meanwhile, compared with that of the *A*_2_–*B*_2_ (120 cm) path, the extra average attenuation of the *A*_3_–*B*_3_ path (180 cm) and the *A*_4_–*B*_4_ path (240 cm) increased by 0.78 dB (measurement) and 1.49 dB (measurement), respectively.

It can be found that the above increments are less than that of the IBC based on a single human body [21,26,27], which can be explained by the fact that, over the same distance, the total impedance of the channel including the handshake and arm in the IBC via handshake channel is less than that of the arm channel in the IBC based on a single human body. Therefore, the impedance corresponding to the unit distance of the channel including handshake and arm is also less than that of the arm channel, which results in the increase in distance along the arm path having less influence on signal attenuation in the IBC via handshake channel. Further research is needed to clarify this issue in future work.

Moreover, according to [23], the transverse impedance corresponding to unit length of a particular human body part decreases with the increase in cross-sectional area, while the cross-sectional area of the torso is generally two orders bigger than that of arm [31]. Therefore, the transverse impedance of torso is far less than that of the arm under the same conditions, which results in the attenuation differences between path *A*_2_–*B*_2_ and path *A*_3_–*B*_3_ being relatively small, while the attenuation differences between *A*_1_–*B*_1_ path and *A*_2_–*B*_2_ path are relatively big [26].

### 4.4. Comparison between Handshake Channel and Single Body

**Figure 15:**
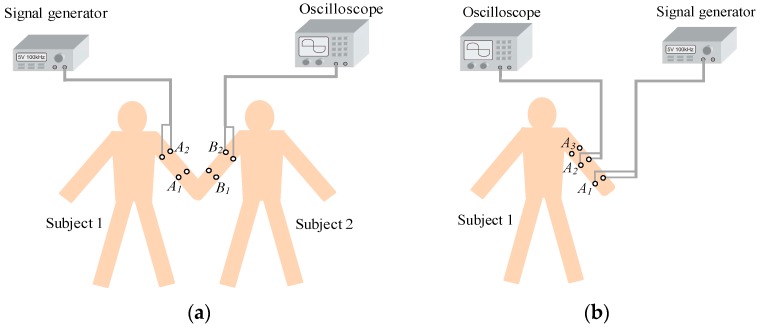
The in vivo measurements of the signal transmission characteristics. (**a**) IBC via handshake channel; (**b**) IBC via single body channel.

To compare the characteristics between the IBC via handshake channel and the IBC via single body channel, in vivo measurements were carried out. As shown in Figure 15a, signal was transmitted along the arms of the two subjects, where the distances were 30 cm (*A*_1_–*B*_1_) and 40 cm (*A*_2_–*B*_2_), respectively. Figure 15b shows the IBC via single body channel, for which the transmitting electrodes were placed on the position of *A*_1_, while the receiving electrodes were placed on *A*_2_ or *A*_3_, resulting in signal transmission distances of 30 cm (*A*_1_–*A*_2_) and 40 cm (*A*_1_–*A*_3_), respectively. Therefore, the difference between Figure 15a,b is the contact impedance (*Z_cp_*) corresponding to the contacting parts caused by shaking hands.

**Figure 16:**
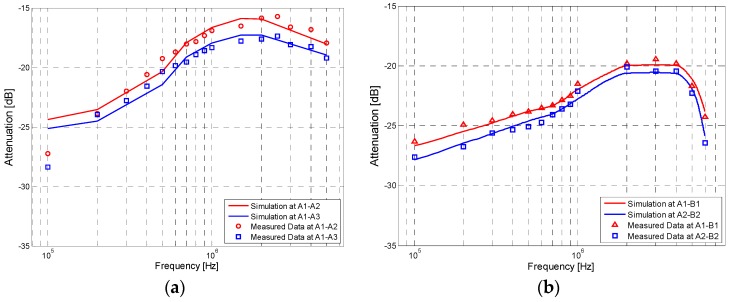
Simulation and measurement results of the galvanic coupling intra-body communication (**a**) Results for the galvanic coupling intra-body communication via handshake channel; (**b**) Results for the IBC via single body channel.

Figure 16 shows the simulation and measurement results of the IBC via handshake channel and the IBC via single body channel. The *K* of Figure 16a is 9.36 dB, while the *K* of Figure 16b is 20.28 dB, which is the value of correction factor for the IBC via single body channel [23]. We can find that, due to the influence of *Z_cp_*, the results of Figure 16a are different from those of Figure 16b, which are described as follows: (1) the signal attenuation of the handshake channel is much bigger than that of the single body channel. According to the simulation results, the maximum and minimum differences between them are 4.80 dB (1.05 MHz, 60 cm) and 1.71 dB (220 kHz, 40 cm), respectively; (2) the peak frequency of the galvanic coupling IBC via handshake channel is within the range of 2–4 MHz, while that of the IBC via single body channel is within the range of 1.5–2.1 MHz; (3) compared with the results for single body, the signal attenuations of the handshake channel decrease more slowly in the frequency range of 100 kHz–2 MHz. In concrete terms, the results of *A*_1_-*B*_1_ decrease 6.97 dB while the corresponding value of *A*_1_–*A*_2_ is 8.44 dB in this range. Meanwhile, the signal attenuations for the handshake channel increase more rapidly in the signal frequency range of 4–6 MHz. For instance, the results of *A*_1_–*B*_1_ increased by 4.61 dB, while the corresponding value of *A*_1_–*A*_2_ was only 2.16 dB in this range.

### 4.5. Influence of the Distance between the Inter-Electrodes

**Figure 17:**
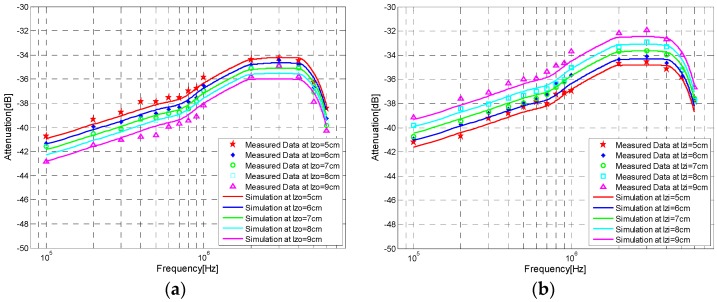
Simulation results and the measurement results corresponding to Z*_i_* and Z*_o_*. (**a**) Results corresponding to different inter-electrodes distances of *Z_o_*, (**b**) Results corresponding to different inter-electrodes distances of *Z_i_*.

In order to investigate the influence of the distance between the inter-electrodes on the galvanic coupling intra-body communication via handshake channel, the corresponding measurements and simulations were carried out. Firstly, the distance between the inter-electrodes of the transmitter, which corresponds to *Z_i_*, was set at 5 cm, 6 cm, 7 cm, 8 cm and 9 cm, respectively, while the distance corresponding to *Z_o_* at the receiver was fixed at 6 cm, and the signal transmission distance was 30 cm. The results are shown in Figure 17a. Then, the distance corresponding to *Z_i_*, was fixed at 6 cm, while the distance corresponding to *Z_o_* was set at 5 cm, 6 cm, 7 cm, 8 cm and 9 cm, respectively. The results corresponding to this case are shown in Figure 17b.

According to the results of Figure 17, when the distances between the inter-electrodes increased from 5 cm to 9 cm, the corresponding decrease of signal attenuations was found, which indicates that the distance between the inter-electrodes does influence the signal attenuation of the galvanic coupling IBC via handshake channel. Meanwhile, the influence of the distance between the inter-electrodes is comparatively small. In concrete terms, according to Figure 17a, an increase of 1 cm distance between the inter-electrodes led to an extra average attenuation of 0.334 dB in the simulation results, while the corresponding value of Figure 17b was 0.343 dB.

## 5. Conclusions

In this paper, a method for modeling the galvanic coupling IBC via handshake channel is proposed, while a corresponding mathematical model is developed. Finally, the validity of the developed model is verified by in vivo measurements. Moreover, the characteristics of the galvanic coupling IBC via handshake channel are compared with those of the IBC via single body channel. Our results indicate that the proposed method will offer significant advantages in the theoretical analysis and application of the IBC via handshake channel.

Some conclusions are, therefore, listed as follows: (1) the simulation results based on the proposed mathematical model basically agree with the corresponding in vivo measurement results; (2) the signal attenuations of the galvanic coupling IBC via handshake channel decrease in the frequency range of 100 kHz–2 MHz, reach their minimum values at 2–4 MHz, and increase rapidly at 4–6 MHz; (3) the signal attenuations of the galvanic coupling IBC via handshake channel are bigger than that of the IBC via single body channel. Meanwhile, the peak frequency of the galvanic coupling IBC via handshake is within 2–4 MHz, and that of the IBC via single body channel is within 1.5–2.1 MHz; (4) the distance between the inter-electrodes influences the signal actuation of the galvanic coupling IBC via handshake channel, while the extra average attenuation corresponding to the increase of 1 cm distance is less than 0.5 dB.

In our future work, the modeling method and the achieved characteristics of the galvanic coupling IBC via handshake channel will be verified by using small battery-operated IBC transmitter and receiver, while the implementation method of the information interaction based on handshake channel will be investigated.

## Figures and Tables

**Table 1:** Parameters of the subjects.

Subject	Age	BMI	Weight/kg	Head/cm	Arm/cm	Hand/cm			Leg/cm	Torso/cm	
				*D_h_*	*D_a_*	*L_hl_*	*L_hw_*	*L_hh_*	*D_l_*	*D_t_*_1_	*D_t_*_2_
Subject A	24	38.67	71	18	10.0	9.2	8.1	2.0	29	30	20
Subject B	23	20.67	67	18	10.2	8.9	8.0	2.0	27	29	20

**Table 2:** Some parameters used in the simulations.

Frequency	*Z_c_*	*Z_At_*_1_/*Z_At_*_2_	*Z_Bt_*_1_/*Z_Bt_*_2_	*Z_i_*	*Z_o_*	*Z_cp_*	*R*_0_	*Z_r_*
0.1 MHz	206 Ω	(534.2 × L) Ω	(505.6 × L) Ω	2083.1 Ω	2088.7 Ω	824 Ω	50 Ω	1 MΩ
	495.0 pF	(0.386/L) nF	(0.406/L) nF	15.10 pF	14.3 pF	111 µF		
1 MHz	126 Ω	(328.1 × L) Ω	(312.5 × L) Ω	1437.9 Ω	1509.8 Ω	532.5 Ω		
	200.5 pF	(0.135/L) nF	(0.141/L) nF	4.69 pF	4.467 pF	405 µF		

**Table 3:** The average and standard deviation of *A*_1_–*B*_1_.

Frequency/MHz	0.1	0.2	0.5	0.7	1	1.5	3	5
Average/dB	−32.32	−30.93	−31.11	−30.11	−28.13	−26.12	−26.44	−32.46
Standard Deviation/dB	0.75	0.87	1.07	1.34	1.37	1.63	0.88	1.40

## Abbreviations

The following abbreviations are used in this manuscript:

IBC	intra-body communication
BSN	body sensor network
